# Supplementary material for: Performance of a Mobile 3D Camera to Evaluate Simulated Pathological Gait in Practical Scenarios
Source: Sensors (Basel). 2023 Aug 4;23(15):6944. doi: 10.3390/s23156944 (PMC10422615; doi:10.3390/s23156944)
Supplement: Supplementary file 1 [file sensors-23-06944-s001.zip › Statistics_SPSS_code/README.html]

Statistics

By Diego Guffanti (ORCID: 0000-0002-1244-291X)

Technologies Used:

1) IBM SPSS Statistics 26

Description:

These scripts allows users to excecute statistical comparison between

matrices of features between ROBOGAIT and Xsens systems.

Instrucctions:

1) Copy the "Statistics\_SPSS\_code" folder to the user desktop.

2) Open the dataset "Dataset\_Robot\_Xsense.sav" file in SPSS. This will

define the file as the file to work with.

3) Open the syntax file "Syntax\_Robot\_Xsense.spss"

4) Go to the main menu, click "run", and then click "all".

5) Alternatively you can directly open the results by opening the file

"Results\_statistics.spv"

6) Syntax contains an example of a comparison for a given feature. To

compare other features just type the name of the feature in the

corresponding line of code.

7) We have included a .doc file containing a summary of results obtained

for the GEE test.

This information is used to evaluate direct differences between the two

systems across all conditions, and to flag significant

differences between pathological and normal walking within systems
