# Supplementary material for: Performance of a Mobile 3D Camera to Evaluate Simulated Pathological Gait in Practical Scenarios
Source: Sensors (Basel). 2023 Aug 4;23(15):6944. doi: 10.3390/s23156944 (PMC10422615; doi:10.3390/s23156944)
Supplement: Supplementary file 1 [file sensors-23-06944-s001.zip › Statistics_SPSS_code/summary_results.pdf]

systems: 1: robot 2: xsens

conditions: 1: normal; 2: CP; 3: MS; 4: AT

	P systems		P Robot normal vs CP	P Robot normal vs MS	P Robot normal vs AT	P xsens normal vs CP	P xsens normal vs MS	P xsens normal vs AT	P systems normal	P systems CP	P systems MS	P systems AT
Stepwidthm	.211		.000	.000	.000	.000	.000	.000	.093	.263	.301	.784
leftstridelengthm	.000	R>x	.000	.032	.000	.000	.002	.000	.000	.000	.000	.000
leftstridetimes	.349		.001	.117	.023	.000	.117	.024	.365	.187	.064	.396
leftsteptimes	.000	R<x	.003	.022	.006	.002	.170	.070	.000	.039	.027	.929
leftcadencestepsmin	.000	R>x	.004	.02	.004	.003	.287	.165	.000	.024	.346	.578
percentageoffootstance	.001	R>x	.003	.000	.001	.247	.047	.037	.000	.023	.205	.219
trunktilt.rom	.000	R<x	.000	.041	.004	.090	.019	.000	.000	.179	.000	.000
pelvistilt.rom	.000	R<x	.000	.342	.425	.001	.001	.000	.081	.035	.000	.000
pelvisrot.rom	.041		.054	.007	.128	.269	.631	.962	.084	.050	.143	.071
hipabdadd.rom	.806		.221	.359	.855	.000	.994	.367	.006	.001	.189	.616
hipflext.rom	.290		.000	.036	.001	.000	.473	.074	.019	.091	.915	.300
kneeflext.rom	.479		.010	.457	.009	.000	.463	.000	.000	.000	.002	.083
hipmax.extensionduringstancedegree	.778		.000	.006	.000	.000	.254	.005	.004	.173	.705	.595
hipmax.flexionduringswingdegree	.053		.000	.718	.104	.000	.658	.747	.820	.000	.516	.990
kneeinitialcontactpositiondegree	.014		.000	.177	.000	.000	.819	.000	.000	.000	.000	.075
kneemax.flexioninloadresponsedegree	.000	R>x	.000	.777	.005	.000	.880	.000	.000	.202	.000	.209
kneepositionattoeoffdegree	.000	R>x	.000	.822	.019	.000	.991	.000	.000	.022	.000	.000
kneemax.flexionduringswingdegree	.095		.000	.831	.384	.004	.036	.005	.552	.154	.001	.000

Significant if p<0.003 (0.05/18)
